# Cell-Wall Glycolipid Mutations and Their Effects on Virulence of *E. faecalis* in a Rat Model of Infective Endocarditis

**DOI:** 10.1371/journal.pone.0091863

**Published:** 2014-03-17

**Authors:** Christoph Haller, Martin Berthold, Dominique Wobser, Andrea Kropec, Marinella Lauriola, Christian Schlensak, Johannes Huebner

**Affiliations:** 1 Department of Thoracic and Cardiovascular Surgery, University Medical Center Tübingen, Tübingen, Germany; 2 Division of Infectious Diseases, Department of Medicine, University Medical Center Freiburg, Freiburg, Germany; 3 Division of Pediatric Infectious Diseases, Dr. von Hauner Children's Hospital, Ludwig-Maximilians-University, Munich, Germany; University of Kansas, United States of America

## Abstract

Enterococci are among the major pathogens implicated in cardiac infections and biofilm formation. *E. faecalis* has been shown to play an important role in infectious endocarditis. Several distinct mechanisms for biofilm formation have been identified in *E. faecalis*. Our group has previously characterized two distinct bacterial glucosyltransferases playing key roles in the production of the major cell wall glycolipids and leading to reduced biofilm production. To assess if this mechanism is involved in the pathogenesis of enterococcal endocarditis we compared the wild-type strain of *E. faecalis* 12030 with two mutants in gene EF2891 and EF2890 respectively in a rat model of infective endocarditis. The results showed less endocarditic lesions and reduced colony counts per vegetation in the two mutants. indicating that the modification of bacterial surface lipids results in significantly reduced virulence in infective endocarditis. These results underscore the important role of biofilm formation in the pathogenicity of enterococcal endocarditis and may indicate an interesting target for novel therapeutic strategies.

## Introduction

The number of multidrug resistant bacteria has increased steadily over the past decades, and the associated problems gain more and more importance with regard to infections, especially in hospitalized patients. Every year the number of patients dying from MRSA-infections in the US almost outnumbers those dying from AIDS, tuberculosis, and viral hepatitis combined [1]. The consequence is not only an enormous rise of health care costs, but also a significantly higher mortality.

Enterococci are currently the second most common cause of nosocomial infections in the US [2]. In Europe, incidences of vancomycin-resistant enterococcal infections are increasing too [3]. *Enterococcus faecalis* is the third most common cause of prosthetic valve endocarditis [4] and despite steadily improved antibiotic strategies the incidence of bacterial endocarditis remained stable throughout the last 40 years.

The ability to produce biofilm plays a crucial role in foreign body infections, such as prosthetic valve endocarditis [5]–[9]. Nevertheless, the underlying mechanisms of biofilm formation and maintenance still need to be further investigated [5], [9], especially in enterococcus where these mechanisms have not been studied as extensively as in staphylococci [10].

Mutants of enterococcal strains with a reduced ability to produce biofilm have been studied previously by several investigators [10] and in vivo bacteremia [11], endocarditis [12] and urinary tract infection models [13] could confirm the reduced pathogenicity of those strains.

In the present study we specifically studied two deletion mutants of *E. faecalis* 12030 unable to produce bacterial glycolipids regarding their ability to cause native valve endocarditis in a rat endocarditis model. The deletion mutants were deficient for glucosyltransferases referred to as biofilm-associated glycolipid synthesis A (Δ*bgsA*) and biofilm-associated glycolipid synthesis B (Δ*bgsB*), respectively. Both enzymes play a crucial role in the biosynthesis of Diglycosyl-diacylglycerol (DGlcDAG) showing accumulation of Monoglycosyl-diacylglycerol (MGlcDAG) in Δ*bgsA* and a complete loss of glycolipids in *ΔbgsB*, as reported previously by Theilacker et al. [11], [14]. Our previous results demonstrated that elimination of glycolipid synthesis results in a reduced accumulation of biofilm mass rather than in impaired adherence itself [11]. Adhesion of enterococci to colon carcinoma cell-line Caco2 was specifically inhibited by up to 47% through the addition of DGlcDAG, suggesting a role of glycolipids in cellular adherence to gastrointestinal epithelia [15]. To assess if this mechanism is also operative in native valve endocarditis we compared theses mutants with the wild type strain using a rat endocarditis model.

## Materials and Methods

### Bacterial strains and growth conditions

The different bacterial strains used are summarized in Table 1. Enterococci were grown at 37°C without agitation in tryptic soy broth (TSB; Carl Roth).

**Table 1:** Bacterial strains.

Bacterial strain	Description	Reference
*E. faecalis* 12030	clinical isolate	[16]
*12030ΔbgsA*	deletion mutant in glycosyltransferase EF2891, no DGlcDAG	[14]
*12030ΔbgsB*	deletion mutant in glycosyltransferase EF2890, no glycolipids	[11]

### Deletion mutant EF2891 (12030ΔbgsA) and EF2890 (12030ΔbgsB)

**Figure 1:**
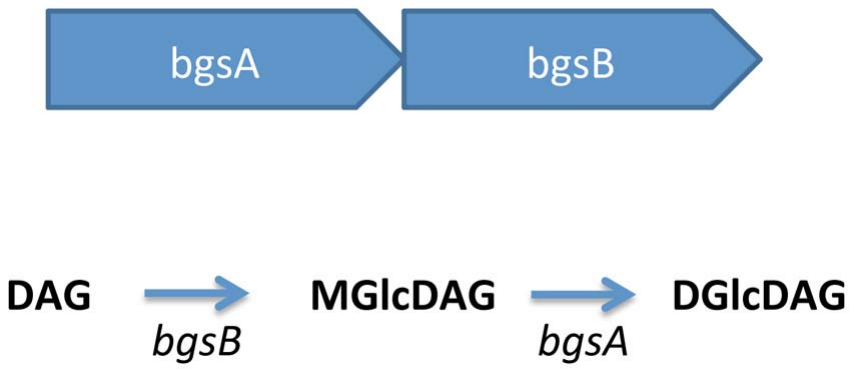
Genetic organization of the bgs-locus and biosynthetic pathway of glycolipid synthesis.

The deletion mutants were derived from a strong biofilm-forming wild-type strain *E. faecalis* 12030 (12030 wt) [16], [17]. The mutant strains are non-polar deletion mutants in the biofilm-associated glycolipid synthesis A and B gene. Mutations were created by targeted mutagenesis and deletion of an internal fragment of 863 bp (12030ΔbgsA) [14] and 790 bp (12030ΔbgsB) as described previously [11], [14]. The deletion of *bgsA* leads to an altered synthesis of cell wall glycolipids lacking diglucosyl–diacylglycerol and overproducing monoglucosyl–diacylglycerol while deletion of *bgsB* leads to a complete loss of cell membrane glycolipids and an altered expression of lipoteichoic acids. Figure 1 shows the organization of the *bgs*-locus and the putative biosynthetic pathway of glycolipid synthesis [11].

### Preparation of inoculum

A dose-response relationship for the colonization of the vegetations was determined for the wild-type strain to ensure a reproducible and comparable bacterial inoculum. Based on preliminary results, we chose inoculation doses between 1–2*10^6^ cfu per animal, which led to a reliable transient bacteremia of at least 24 h. To determine absolute values of inoculated bacteria and to compare different inocula, serial dilution and cfu counts of the inocula were done.

### Rat model of endocarditis

The rat model of endocarditis is based on experimental findings of colonization of non-bacterial thrombotic endocarditis in rabbits by Durack and Beeson in 1972 [18]. This technique was then transferred to a rat model in 1978 by Santoro and Levision [19].

**Figure 2:**
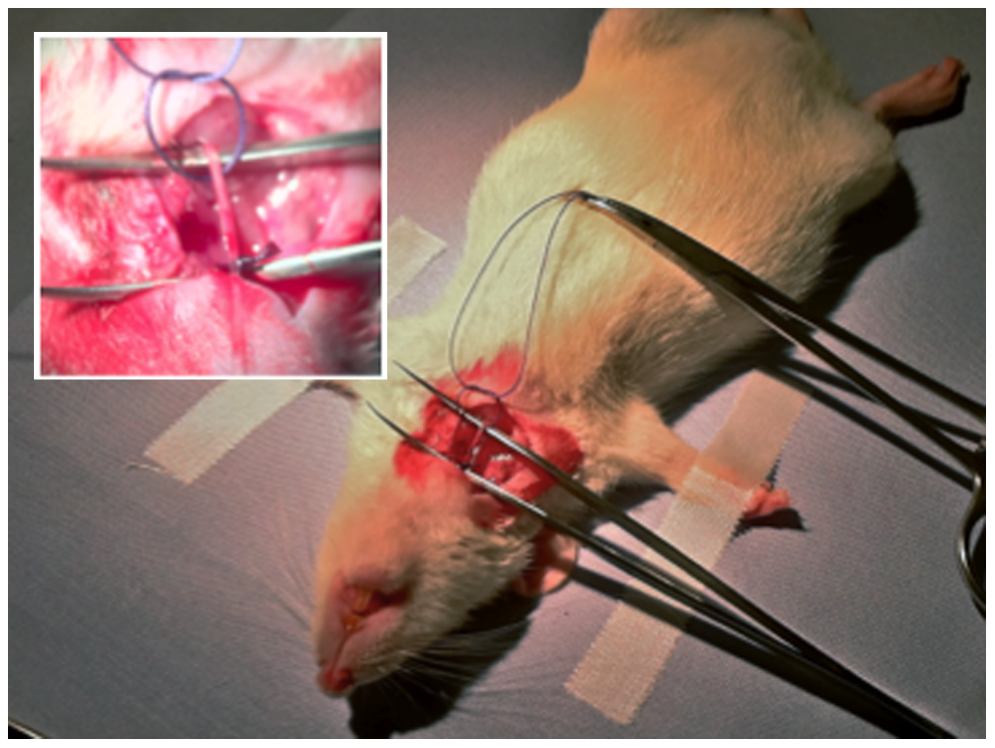
Intraoperative view with catheter placed in the right carotid artery.

Female Wistar rats (Charles River Laboratories Germany GmbH), weighing 200 to 300 g were used. The animals were anesthetized by subcutaneous application of 5.75% ketamine and 0.2% xylazine. Nonbacterial thrombotic endocarditis was caused by insertion of a small plastic catheter (polyethylene tubing; Intramedic PE 10) via the right carotid artery (Figure 2). The artery was accessed by cutting the neck laterally on the right side. The carotid artery could be easily exposed and ligated distally. The polyethylene catheter was introduced via a small incision of the vessel and advanced until a slight resistance indicated passage through the aortic valve. The catheter was advanced through the aortic valve into the left ventricle. Proper placement was ensured via invasive pressure measurement through the catheter's lumen. To minimize confounding factors we focused on standardization of the catheter insertion and its positioning. Preliminary experiments without secondary bacterial colonization showed that pressure monitoring and therewith objectification of catheter positioning could minimize overly traumatic injury (which may lead to pronounced thrombotic lesions) and ensured constant lesions. The catheter was secured in place and distally ligated. A simple running suture was used for wound closure. Postoperatively the rats were returned to their cage and monitored closely.

Inoculation of bacteria followed 48 h after catheter placement via injection into the tail vein. Ten rats per study group were randomly assigned to two groups, one receiving the wild-type strain 12030 wt, and the other being challenged with the deletion mutant 12030ΔbgsA. In a separate series (n = 10) groups were divided using 12030wt and 12030ΔbgsB respectively. Animals were sacrificed on postoperative day 6 and the correct placement of the catheter was verified. The extent of native valve endocarditis was assessed and graded macroscopically with an objective grading system as outlined in table 2. Subsequently valve vegetations were removed aseptically. After weighing of the vegetations, 500 μL TSB was added per sample and the vegetations were homogenized on ice using a tissue homogenizer. The homogenizer was cleaned with ethanol and flamed after each homogenization. Serial dilutions of the samples were made in TSB and plated. Quantitative assessment was performed by weighing of the vegetations as well as culturing serial dilutions on agar plates incubated over night at 37°C.

**Table 2:** Macroscopic grading system.

grading	description
**1**	no visible deposits on valve
**2**	isolated deposits on valve, but unclear if thrombotic or endocarditic in nature
**3**	isolated endocarditic vegetations
**4**	multiple scattered endocarditic vegetations
**5**	confluent endocarditic vegetations covering the valve leaflets
**6**	bold endocarditic vegetations covering valve apparatus without further extent
**7**	vegetations on valve with circumscribed extent
**8**	fulminant vegetations on valve, further extension into left ventricular outflow tract and aortic root restricted to catheter
**9**	fulminant vegetations on valve extending into left ventricular outflow tract and aortic root
**10**	fulminant vegetations on valve extending into left ventricular outflow tract and aortic root and involvement of ventricular endocardium

### Statistical analysis

Comparisons of groups were performed using IBM SPSS Statistics Desktop 20.0 and GraphPad Prism 5.04 for Microsoft Windows. The primary evaluation criterion was the bacterial count in the vegetation (cfu per gram and per ml, respectively). The mean and standard deviation was calculated for each group. Statistical significance was determined by Mann-Whitney U test.

### Ethics statement

All animal experiments were performed with permission of the regional administrative authority Freiburg (animal welfare committee of the University of Freiburg; Regierungspräsidium Freiburg Az 35/9185.81/G-07/72) and in accordance with the German animal protection law (TierSchG). The rats were handled in accordance with good animal practice as defined by FELASA and the national animal welfare body GV-SOLAS.

## Results

### Comparison of 12030wt with 12030ΔbgsA and with 12030ΔbgsB

**Figure 3:**
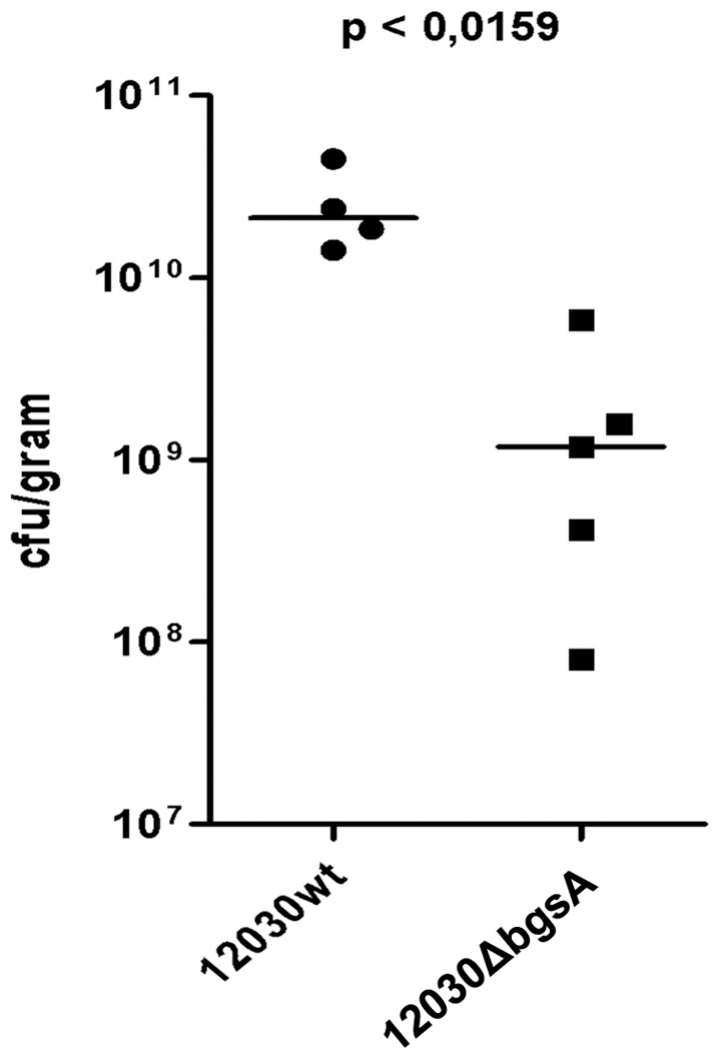
Comparison of virulence of *E. faecalis* wild type and 12030ΔbgsA specified by CFU/gr (bar indicates median).

Mutant 12030ΔbgsA showed a significantly reduced rate of endocarditis (figure 3) compared to wild-type bacteria. The inocula used had effective concentrations of 1.225×10^6^ cfu/ml for 12030 wt and 2.325×10^6^ cfu/ml for 12030ΔbgsA.

One rat of the wild-type group died of unknown cause directly after catheter placement. Therefore 12030 wt vs. 12030ΔbgsA groups finally consisted of 4 rats infected with *E. faecalis* 12030 wt and 5 rats infected with *E. faecalis* 12030ΔbgsA. Rats infected with 12030ΔbgsA showed considerably but insignificantly less severe endocarditic lesions macroscopically (p<0.39; table 3). Bacterial vegetations of 12030ΔbgsA contained significantly less CFU per milliliter and per gram compared to those formed by 12030 wt using the Mann–Whitney U test. The average colony count for rats infected by 12030 wt and 12030ΔbgsA respectively were 2.55×10^10^±1.37×10^10^ and 1.82×10^9^±2.33×10^9^ CFU/gr, p<0,016 (figure 3).

**Table 3:** Macroscopic grading and absolute weight of vegetation of endocarditic lesions of 12030wt and 12030ΔbgsB, showing insignificantly higher grades (p<0.39) and weights in the wild type (p<0.35).

No.	strain	macroscopic grade	weight of vegetation (mg)
**1**	12030 wt	7/10	3,56
**2**	12030 wt	7/10	3,35
**3**	12030 wt	8/10	9,54
**4**	12030 wt	3/10	1,59
**5**	12030 wt	†	†
**6**	12030ΔbgsA	2/10	0,40
**7**	12030ΔbgsA	8/10	3,25
**8**	12030ΔbgsA	5/10	2,60
**9**	12030ΔbgsA	4/10	3,35
**10**	12030ΔbgsA	5/10	1,42

**Figure 4:**
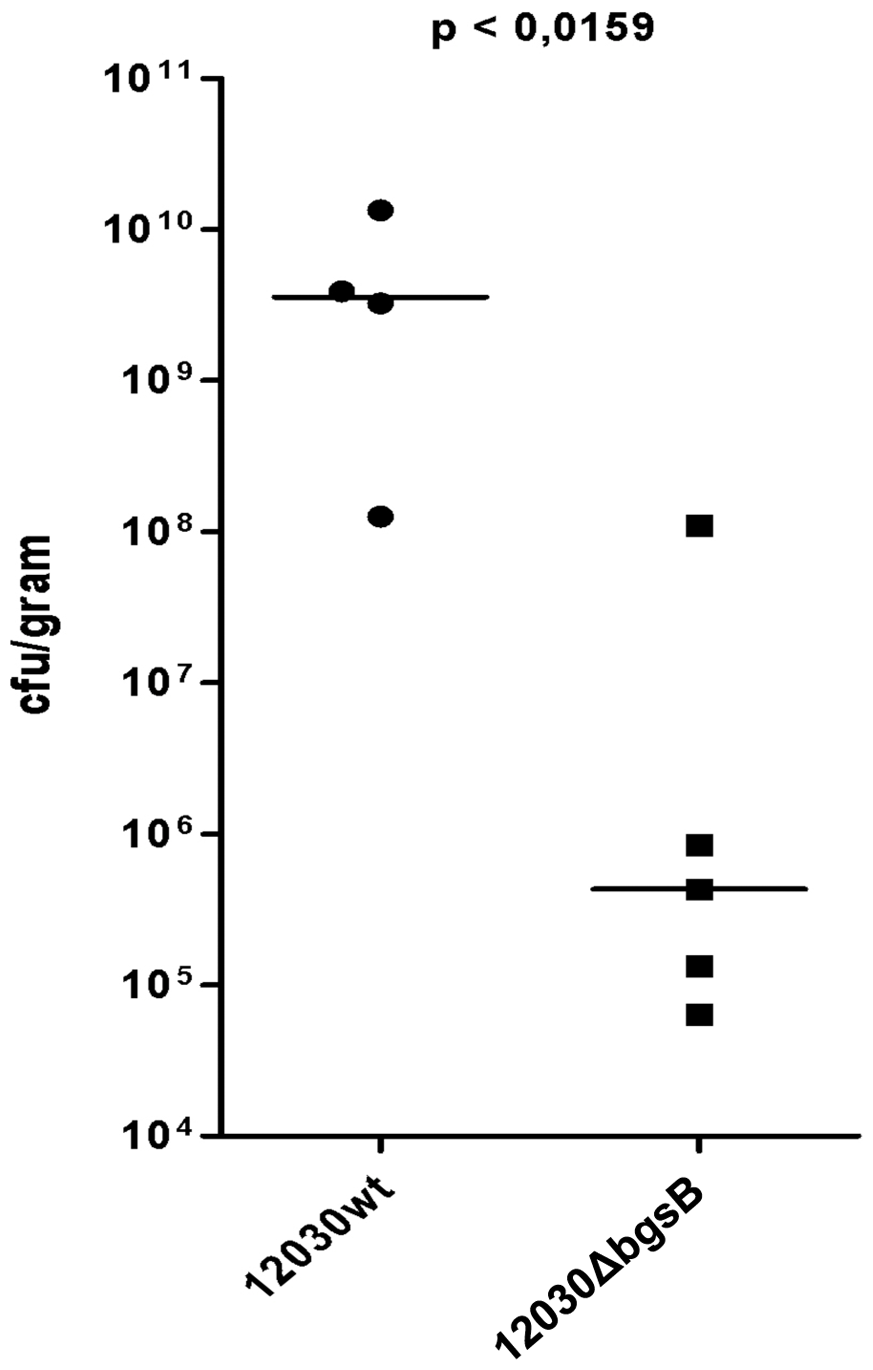
Comparison of virulence of *E. faecalis* wild type and 12030ΔbgsB specified by CFU/gr (bar indicates median).

Mutant 12030ΔbgsB also showed a significantly reduced rate of endocarditis compared to wild-type bacteria (Figure 4). The final inocula used in this experiment showed 2.1×10^6^ cfu/ml for 12030 wt and 1.82×10^6^ cfu/ml for 12030ΔbgsB. Again one rat of the wild-type group died during placement of the catheter. Thus the wild-type group consisted of 4 rats whereas the 12030ΔbgsB group counted 5 rats. Endocarditic lesions were graded macroscopically and showed significantly less severe vegetations in rats challenged with 12030ΔbgsB compared to rats inoculated with 12030 wt (p<0.39; table 4). Bacterial vegetations of 12030ΔbgsB contained less CFU compared to those by 12030 wt. Statistical significance was tested using the Mann–Whitney U test. The average colony count in rats infected with 12030 wt and 12030ΔbgsB respectively were 5.16×10^9^±5.72×10^9^ and 2.24×10^7^±4.93×10^7^ CFU/gr, p<0.016 (figure 4).

**Table 4:** Macroscopic grading and absolute weight of vegetation of endocarditic lesions of 12030wt and 12030ΔbgsB, showing significantly higher grades (p<0.05) and weights in the wild type (p<0.05).

No.	strain	macroscopic grade	weight of vegetation (mg)
**1**	12030 wt	9/10	9,76
**2**	12030 wt	7/10	5,14
**3**	12030 wt	5/10	3,7
**4**	12030 wt	7/10	8,93
**5**	12030 wt	†	†
**6**	12030ΔbgsB	1/10	0,44
**7**	12030ΔbgsB	1/10	1,04
**8**	12030ΔbgsB	2/10	0,58
**9**	12030ΔbgsB	1/10	0,78
**10**	12030ΔbgsB	2/10	0,71

## Discussion

Enterococcal infections are an increasing clinical problem worldwide [2]. While enterococci certainly are not as virulent as other gram-positive cocci (such as *Staphylococcus aureus* or *Streptococcus pyogenes*), they often exhibit broad-spectrum resistance to antimicrobials, are able to acquire antibiotic resistance traits via elaborate molecular mechanisms, and are able to resist harsh environmental conditions [20]. Biofilm formation has been shown to play a crucial role, especially in endocarditic lesions and catheter-associated infections [21]. A variety of biofilm-associated genes have been described in enterococci, encoding adhesins, autolysins, polysaccharides, glycolipids and other molecules, each of these causing virulence in specific settings [10].

The deletion mutant *12030ΔbgsA* is characterized by a loss of the major bacterial glycolipid DGlcDAG and by an accumulation of its precursor, MGlcDAG. Mutant *12030ΔbgsB* completely lacks glycolipids in its cell wall. In a rat model of infective endocarditis we could demonstrate that these two different enterococcal deletion mutants with their altered biofilm formation capabilities produced less endocarditic lesions when compared to the wild type. Despite slightly higher bacterial inocula for the mutant strains, the colony counts in vegetations were significantly reduced both for *12030ΔbgsA* as well as for *12030ΔbgsB* compared to the wild type. We have previously shown that the replacement of DGlcDAG with MGlcDAG leads to a significantly impaired biofilm production in vitro (i.e. on polystyrene plates and colon epithelial cells) and in vivo in a mouse sepsis model [11], [14]. On the other hand, cell morphology, autolysis, growth rate or expression of cell wall-associated proteins were not altered in these mutants [11], [14]. The role of glycolipids in vivo has already been assessed in mouse bacteremia models and a reduced persistence of bacteria in blood, liver, kidneys and spleens has been documented [11], [14]. However, these models do not represent typical biofilm infections.

Certainly our results have to be interpreted cautiously. First of all the small sample size weakens the results with regard to statistical power, although our experience with this model of infective endocarditis taught us that results are reproducible. Furthermore the extent of non-infective thrombotic endocarditis plays a crucial role in the secondary bacterial colonization. To ensure constant non-infective lesions, we assessed the proper placement of the catheter and assessed the lesions themselves in preliminary tests.

Since we did not measure blood stream survival of the inoculated bacteria, the influence of direct bloodstream clearance or the increased sensitivity of the mutants to the innate immune functions of the animals cannot be differentiated. Nevertheless previous data from our group show that the deletion of glucosyltransferase bgsB has no effect on resistance to complement, antimicrobial peptides, and opsonophagocytic killing [11]. The bgsA deletion mutant showed higher susceptibility to opsonophagocytic killing, but comparable sensitivity to complement-mediated killing [14]. Although the significantly reduced endocarditic lesions might be a direct effect of impaired biofilm accumulation on the heart valve itself our results cannot clarify if the reduced virulence of the mutant strains is caused by interaction with host defense instead.

In addition we could identify a putative virulence factor for enterococcal endocarditis, which might offer new therapeutic approaches. Guo et al. for example reported the use of antisense oligodeoxyribonucleotides as a way of gene silencing in *Streptococcus mutants* as a novel approach to decrease biofilm formation [22]. Specific small molecule inhibitors of genes essential for virulence but not directly toxic for bacteria may exert less selective pressure to develop resistances and may be therefore attractive novel therapeutic approaches [23]. Our results indicate that the model presented here is suitable to assess specific virulence factors of enterococcal strains involved in biofilm infections. This will allow the detection and confirmation of additional virulence factors and may lead to the development of novel therapeutic approaches. Further studies are needed to differentiate the factors influencing the formation of bacterial endocarditis in biofilm deficient mutants of enterococci.
